# Structural Investigations of α-MnS Nanocrystals and Thin Films Synthesized from Manganese(II) Xanthates by Hot Injection, Solvent-Less Thermolysis, and Doctor Blade Routes

**DOI:** 10.1021/acsomega.1c02907

**Published:** 2021-10-11

**Authors:** Abdulaziz M. Alanazi, Paul D. McNaughter, Firoz Alam, Inigo J. Vitorica-yrezabal, George F. S. Whitehead, Floriana Tuna, Paul O’Brien, David Collison, David J. Lewis

**Affiliations:** † Department of Chemistry, University of Manchester, Oxford Road, Manchester M13 9PL, U.K.; ‡ Department of Materials, University of Manchester, Oxford Road, Manchester M13 9PL, U.K.

## Abstract

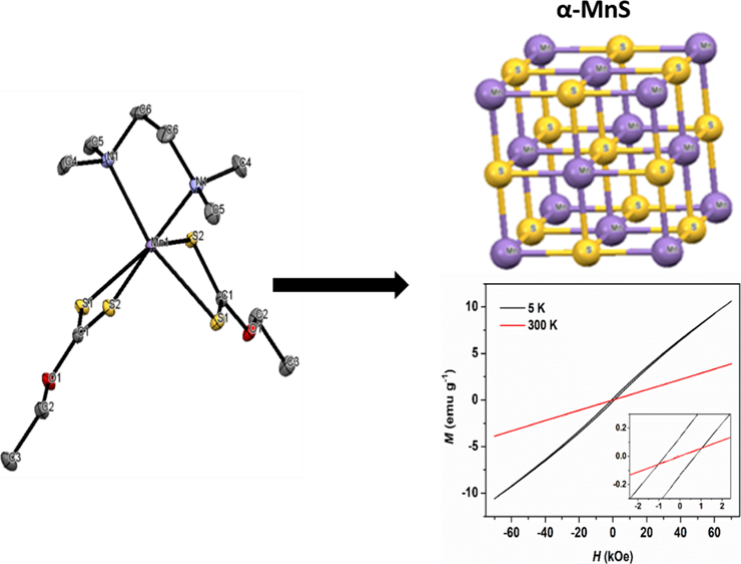

Manganese(II) xanthate complexes of the form [Mn(S_2_COR)_2_(TMEDA)], where TMEDA = tetramethylethylenediamine and R = methyl (**1**), ethyl (**2**), *n*-propyl (**3**), *n*-butyl (**4**), *n*-pentyl (**5**), *n*-hexyl (**6**), and *n*-octyl (**7**), have been synthesized and structures elucidated using single-crystal X-ray diffraction. Complexes **1**–**7** were used as molecular precursors to synthesize manganese sulfide (MnS). Olelyamine-capped nanocrystals have been produced *via* hot injection, while the doctor blading followed by thermolysis yielded thick films. Free-standing polycrystalline powders of MnS are produced by direct thermolysis of precursor powders. All thermolysis techniques produced cubic MnS, as confirmed by powder X-ray diffraction, scanning electron microscopy, energy-dispersive X-ray spectroscopy, and Raman spectroscopy. Magnetic measurements reveal that the α-MnS nanocrystals exhibit ferromagnetic behavior with a large coercive field strength (*e.g.,* 0.723 kOe for 6.8 nm nanocrystals).

## Introduction

**Figure 1:**
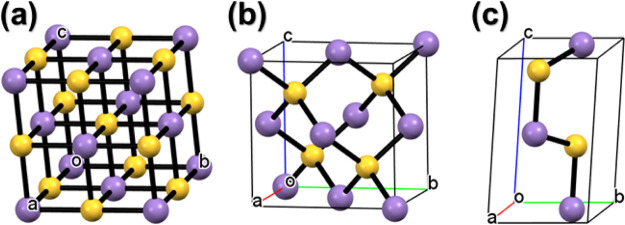
Crystal structures of (a) cubic rock salt (RS) α-MnS, *a*, *b*, *c* = 5.224 Å (ICDD 01-089-4952), (b) metastable cubic zinc blende (ZB) β-MnS, a, b, c = 5.615 Å (ICDD 00-040-1288), and (c) hexagonal wurtzite (WZ) γ-MnS structures, *a* and *b* = 3.979 Å and *c* = 6.446 Å (ICDD 00-040-1289). Color code: Mn, violet; S, yellow. Adapted from ref (8), with permission from Elsevier.

Nanometric manganese(II) sulfide is an attractive material to target as it possesses useful optical, electrical, and magnetic properties that vary in the nanometer size domain, that is, below 100 nm.^1^ Manganese sulfide (MnS) is a wide-band-gap (*E*_g_ ∼ 2.7–3.7 eV) p-type semiconductor which exists in three polymorphs: α, β, and γ; see Figure 1. α-MnS exists in a cubic rock salt and is stable, the β form is metastable with a cubic zinc blende structure, and the γ phase possesses a metastable hexagonal wurtzite structure. Both β-MnS and γ-MnS can be transformed to the rock salt phase by use of high temperature or high pressure.^2^ Possessing both electronic and magnetic properties allows MnS to have potential uses in light-emitting and optoelectronic devices,^3^ magneto-optical devices, and as electrodes in lithium ion batteries.^4,5^

At room temperature, all the three bulk phases, α, β, and γ, are paramagnetic (PM) and are antiferromagnetic (AFM) below their respective Neél temperatures of 154, 100, and 80 K^6^ due to the antiferromagnetic coupling of the high-spin d^5^ Mn^2+^ (*S* = 5/2). With particle sizes decreasing beneath 100 nm, α-MnS displays decreasing *T*_N_, which was attributed to a mixture of PM and AFM phases in the nanocrystals (NCs).^7^

A range of synthetic methods have been used to produce MnS nanostructures including chemical bath deposition, hydrothermal, microwave, solvothermal, and sonochemical methods.^9−14^ Synthetic routes for the controlled synthesis of single-phase MnS NCs have also been reported.^15−17^ For example, Hyeon *et al.*, synthesized hexagonal MnS by heating a mixture of sulfur and MnCl_2_ in oleylamine at 280 °C.^15^ Moreover, the use of Mn(II) dithiocarbamate complexes in the production of manganese sulfide has been reported in a study that examined the effect of the counter anion upon the morphology and phase of the synthesized product.^18^

Metal xanthates are a readily prepared class of precursors that offer a route to a wide variety of metal chalcogenide nanometric materials.^19−23^ Compared to the classic metal dithiocarbamate single-source precursors, metal xanthates thermally decompose at lower temperatures by the Chugaev elimination.^24^ The biproducts formed are SCO, H_2_S, and the corresponding alkene, which are either gasses or volatile liquids, thus lowering the opportunity for contamination of the metal chalcogenide phase formed. Size control over the resulting NCs produced has been observed when increasing the alkyl chain length of the metal xanthate, avoiding the need for additional surfactants.^25,26^

In this work, the novel bis(*O*-alkylxanthato) manganese(II) complexes (alkyl = Me, Et, ^*n*^Pr, ^*n*^But, ^*n*^Pen, ^*n*^Hex, and ^*n*^Oct) stabilized by the bidentate N-donor ligand tetramethylethylenediamine (TMEDA) are synthesized and their crystal structures determined. The complexes are used to produce MnS by three methods: (i) hot injection using oleylamine (OLA) as a capping agent and trioctylphosphine (TOP) as the dispersion medium, (ii) solventless thermolysis to produce free-standing MnS powders, and (iii) doctor blade for deposition of MnS films.

## Results and Discussion

**Figure 2:**
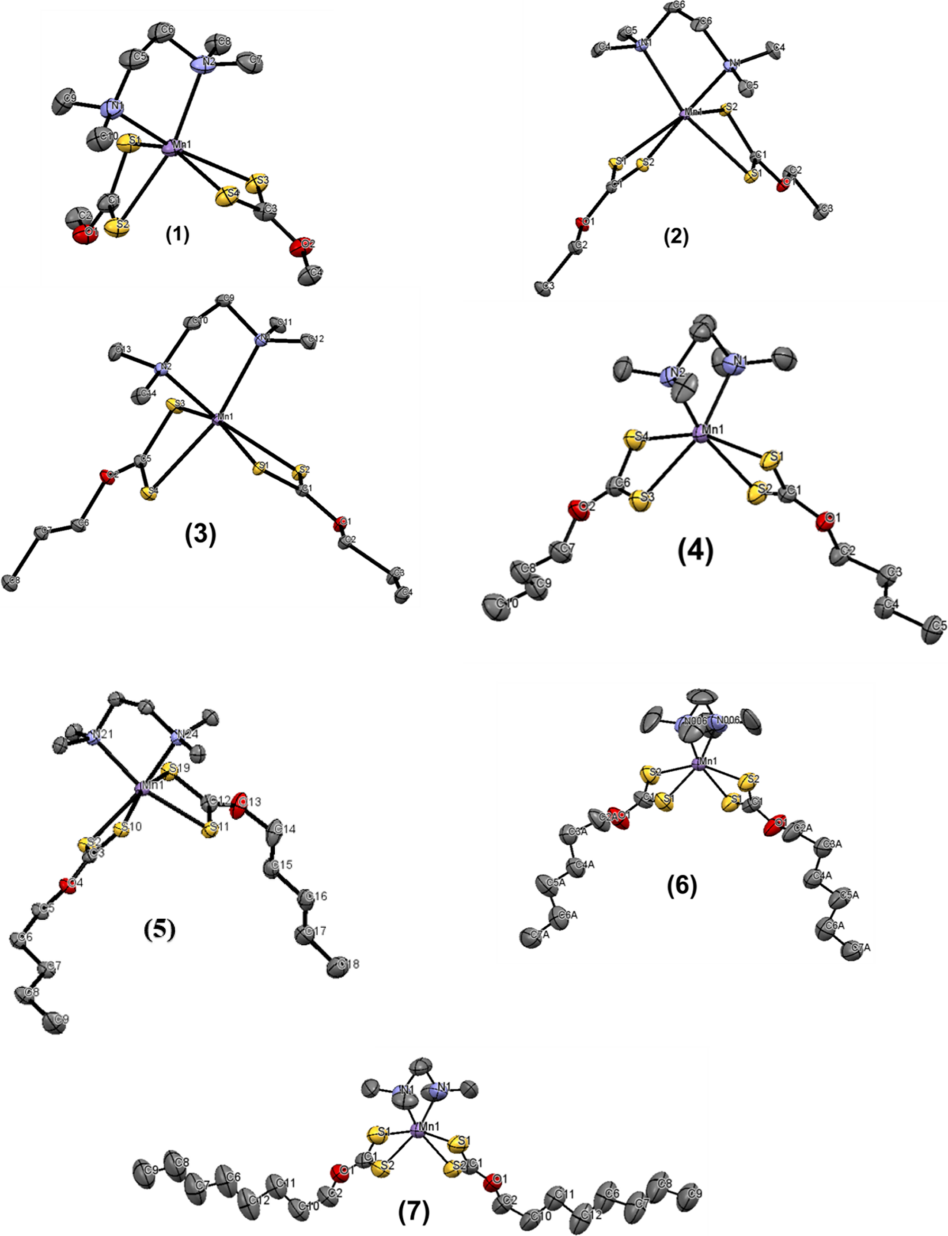
Molecular structures of [Mn(S_2_COMe)_2_·TMEDA] (**1**), [Mn(S_2_COEt)_2_·TMEDA] (**2**), [Mn(S_2_CO^*n*^Pr)_2_·TMEDA] (**3**), [Mn(S_2_CO^*n*^But)_2_·TMEDA] (**4**), [Mn(S_2_CO^*n*^Pen)_2_·TMEDA] (**5**), [Mn(S_2_CO^*n*^Hex)_2_·TMEDA] (**6**), and [Mn(S_2_CO^*n*^Oct)_2_·TMEDA] (**7**). H atoms are omitted for clarity. Violet = Mn, yellow = S, red = O, blue = N, and gray = C. (CCDC 1959720–1959726).

We report here the synthesis and single-crystal structures of six novel manganese xanthates: [Mn(S_2_COMe)_2_·TMEDA] (**1**), [Mn(S_2_COEt)_2_·TMEDA] (**2**), [Mn(S_2_CO^*n*^Pr)_2_·TMEDA] (**3**), [Mn(S_2_CO^*n*^But)_2_·TMEDA] (**4**), [Mn(S_2_CO^*n*^Pen)_2_·TMEDA] (**5**), and [Mn(S_2_CO^*n*^Hex)_2_·TMEDA] (**6**). [Mn(S_2_CO^*n*^Oct)_2_·TMEDA] (**7**) was also prepared and was characterized only by X-ray diffraction (XRD) analysis, elemental analysis, and melting point. These complexes were prepared from the reaction of a previously prepared potassium alkylxanthate and manganese(II) acetate tetrahydrate with the subsequent addition of TMEDA. All the complexes were soluble in common organic solvents such as chloroform, tetrahydrofuran (THF), and toluene. The complexes were stored at −20 °C to prevent decomposition. The structures of the complexes are shown in Figure 2. **2**, **3**, **4**, **6**, and **7** adopt monoclinic crystal systems with space groups *C*2/*c*, *P*2_1_/*c*, *P*2_1_/*c*, *I*2/*a*, and *I*2/*a*, respectively, while **1** is orthorhombic *Pbca* and **5** is triclinic *P*1̅.

In all cases, the central Mn ions were coordinated by six atoms, bound by two xanthate ligands and single TMEDA ligand, with N and S donors arranged in a distorted octahedron. Furthermore, no differences in Mn–S or Mn–N bond distances were observed within the structures of **2**, **6**, and **7**; therefore, the ligands were considered to be in a symmetric (isobidentate) mode. However, the Mn–N bond distances in **5** were significantly different, and a relatively small difference was observed in the cases of **1**, **3**, and **4**; therefore, the ligands were considered to be in an asymmetric mode, as presented in Table S1.

The molecular structure of **2**, [Mn(S_2_COCH_2_CH_3_)_2_·(TMEDA)], is shown in Figure 2, and the selected geometric parameters are presented in Table S2. The shorter Mn–S and the longest Mn–S bond lengths involving the xanthate ligands were 2.5645 and 2.6750 Å, respectively, as listed in Table S1, and were in good agreement with those reported for other analogous 1:1 adducts of Mn-dithiocarbonato (xanthate) complexes.^27^

The symmetric mode of coordination of the xanthate ligands was reflected in the near equivalence of the associated C–S bond distances. Within each of the xanthate ligands, the shorter Mn–S bond had the S atom approximately trans to a N atom, and the two S atoms forming the longer Mn–S bonds were approximately trans to each other. Another difference between the structures was that the shortest Mn–N bond distance was observed in complex **6**, and thus, the N–Mn–N angle was the shortest angle compared with other complexes.

As the alkyl chain length increased in the structures with identical binding of the two xanthates to the Mn, the difference in the bonding modes of the two ligands became more obvious. The difference in the Mn–S bond distances in the symmetrical binding decreased with an increase in the length of the alkyl chain. The Δ(Mn–S) = (longer Mn–S bond distance – shorter Mn–S distance) values for the remaining ligand were 0.11, 0.08, and 0.03 Å for **2**, **6**, and **7**, respectively. In contrast, the difference in the Mn–S bond distances in the asymmetric binding of **1**, **3**, **4**, and **5** displayed no trend with increase in the alkyl chain length.

The relatively short C–O bond distances of 1.333 (2) Å for one ligand in complexes **1**, **2**, **3**, **4**, and **5** were almost the same. However, in **6** and **7**, the C–O bond distances were 1.361 (5) and 1.341 (9) Å, respectively, which were longer than those of the other complexes. The data shown in Table S1 are consistent with a significant contribution of the resonance form of the xanthate anion that features a formal C=O bond and the negative charges on each of the S atoms. In the case of compound **2**, the bidentate N-donor ligands had the same Mn–N1 and Mn–N2 distances (2.293 (15) Å). Because of the restricted ligand bite, the angles N–Mn–N and S–Mn–S were lower than 90° in a regular octahedron. The N–Mn–N angles averaged at approximately 79.22° (8) and S–Mn–S angles at 69.10° (15), as shown in Table S1. The molecular structures of other novel complexes are shown in Figure 2, and selected bond distances and angles are given in Table S1.

The most distinct difference between these compounds was how the ligand frameworks and the presence of hydrogen bonds affected the crystal packing in the extended solid state for all of these complexes. As shown in Figure S1, all the complexes displayed intermolecular hydrogen bonds through the sulfur atoms of the neighboring molecules (C–H···S), while in the complex **4**, the C–H···S interaction linked a molecule with another one by the H from the TMEDA, except complex **5**, wherein the (C–H···S) interaction was not observed. The distances of these interactions were slightly shorter than the sum of the contact radii (van der Waals radii),^28^ as shown in Table S3.

Furthermore, **2**, **3**, and **6** had two main modes of association between molecules; one of them was the H from the adduct contact with the S from the other molecule (N–C–H···S) and the H from the alkyl group contact with S from the other molecule (C–C–H···S), as shown in Figure S1. In contrast, **1** and **7** had one mode of association between molecules, which was the (N–C–H···S) interaction in **1** and the (C–C–H···S) interaction in complex **7**. The complex **5** also exhibited interchelate distances between S from the molecule and S from another molecule (3.491 Å).

**Figure 3:**
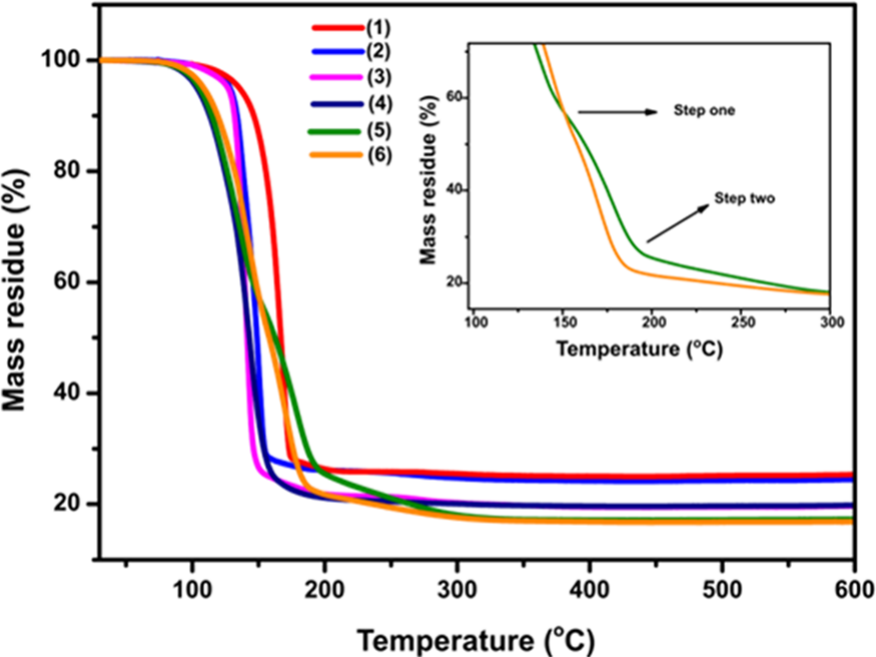
TGA profiles of complexes (**1**–**6**).

Thermal analyses of all the complexes were conducted up to 600 °C under nitrogen; see Figure 3. Complexes **1**, **2**, **3**, and **4** showed a one-step decomposition to form MnS, with the decomposition profile shifting to lower temperature with increase in the precursor alkyl chain length. For chain lengths of pentyl and above, the thermogravimetric analysis (TGA) profiles displayed a two-step breakdown. In the case of **5** and **6** precursors, the mass residue obtained from the TGA profiles for the first decomposition stage (57.5%) agrees with the theoretical value calculated for the removal of one molecule of xanthate and half from a second (58%). All the six complexes gave the final solid residue amounts that matched with the predicted value for MnS; see Table S4.

### MnS NCs Using the Hot-Injection Method

**Figure 4:**
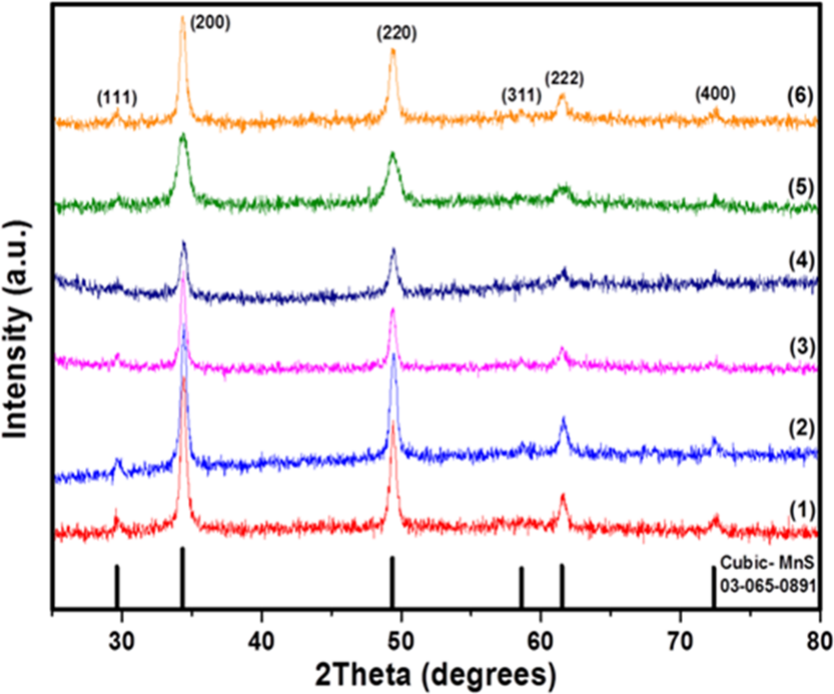
XRD patterns of MnS prepared at 250 °C *via* hot injection from precursors **1**–**6**. The standard pattern (black sticks) is cubic α-MnS (ICDD no. 03-065-0891).

MnS NCs were synthesized using hot injection of manganese alkylxanthate in TOP and then injected into preheated OLA at 230 °C. OLA is used as a coordinating agent and also can catalyze the degradation of complexes at lower temperatures than the other methods.^29,30^ It was observed that complex **2** showed partial decomposition at 200 °C, leading to poor crystallinity of the material produced; see Figure S3. 250 °C yielded a material with good crystallinity, as shown by the *p*-XRD measurements, Figure 4, with reflections corresponding to cubic α-MnS (JCPDS 03-065-0891). The diffraction patterns showed significant changes in the intensity of peaks depending on the chain length. For complexes from **2** to **4**, the fall in intensity with the precursor chain length was greater in the (220) plane.

Estimated crystallite sizes using the Scherrer equation were found to be 19.5, 17.8, 17.0, 14.9, 10.0, and 9.2 nm for MnS NCs from precursors **1**, **2**, **3**, **4**, **5**, and **6**, respectively; see details in Supporting Information. Kan *et al.* have successfully synthesized cubic α-MnS NCs of different sizes ranging from 20 to 80 nm by a colloidal synthesis route through the reaction of MnCl_2_ and S[Si(CH_3_)_3_]_2_ in trioctylphosphineoxide.^7^ The calculated lattice parameters and volume are given in Table S5.

The atomic percentages of the samples prepared by hot injection were determined by energy-dispersive X-ray (EDX) spectroscopy, showing the composition to be approximately 50:50 of Mn and S and suggesting that manganese sulfide was formed (Supporting Information Figure S4, Table S5†).

**Figure 5:**
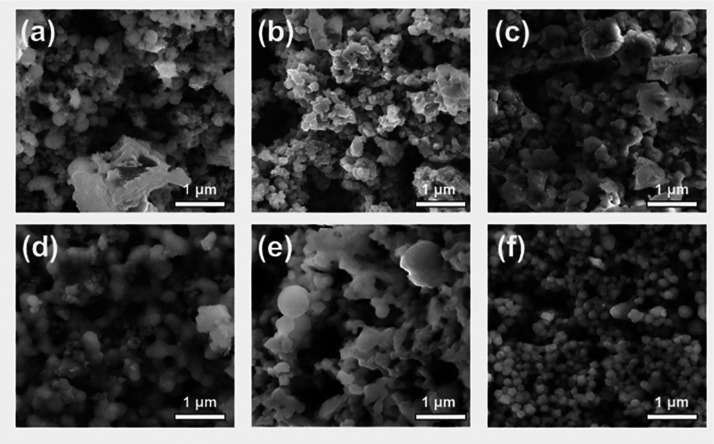
Representative secondary electron SEM images (10 kV) of MnS samples prepared using precursors (a–f) (**1**–**6**) prepared by the hot injection method at 250 °C.

The morphology of the synthesized MnS NCs was observed by scanning electron microscopy (SEM), and the images are shown in Figure 5. The MnS NCs obtained from **1**–**5** have irregular shapes (Figure 5a–e), while those obtained from complex **6** have a more regular morphology across the sample (Figure 5f).

Raman spectroscopy revealed that the MnS synthesized *via* hot injection from precursor **1** exhibited a single band at 635.89 cm^–1^ and remains approximately the same for precursors **2**, **3**, **4**, **5**, and **6**, which is in agreement with reported values; see Figure S5 and Table S5.^31^

### MnS NCs Using Solventless Thermolysis

At lower temperatures of 250 and 300 °C, the complete conversion of **2** [Mn(S_2_COEt)_2_·(TMEDA)] had not occurred when observed by XRD (Figure S7), whereas at 350 °C, good crystallinity demonstrated that complete conversion occurred.

**Figure 6:**
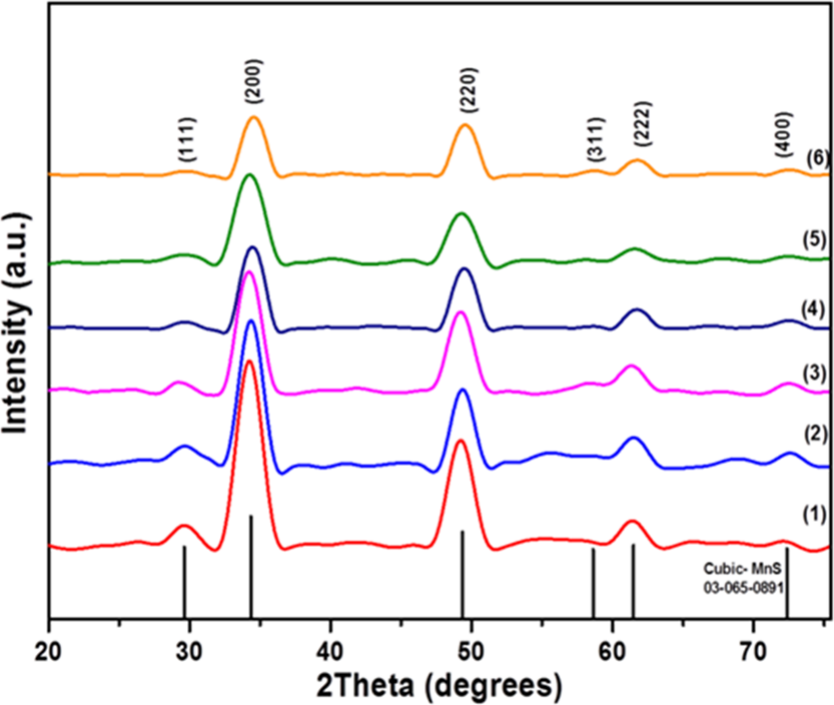
*p*-XRD patterns of MnS prepared at 350 °C *via* solventless thermolysis of precursors (**1**–**6**). The standard pattern is cubic manganese sulfide, MnS (ICDD no. 03-065-0891).

With heating for 1 h at 350 °C, all six precursors (**1–6**) generated MnS, with patterns that are in good agreement with the cubic α-MnS phase (JCPDS 03-065-0891, Figure 6). Crystallite size was estimated with the Debye–Scherrer equation to be 8.2, 6.8, 6.3, 8.9, 7.6, and 8.7 nm for NCs obtained from **1**, **2**, **3**, **4**, **5**, and **6**, respectively (Table S6).

**Figure 7:**
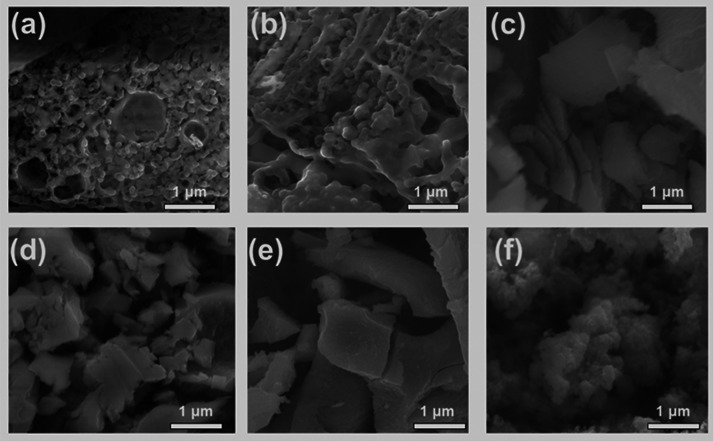
Representative secondary electron SEM images (10 kV) of MnS samples produced using precursors (a–f) (**1**–**6**) and prepared by solventless thermolysis at 350 °C.

For precursors **1** and **2**, the NCs were found to be aggregates consisting of numerous particles (Figure 7a,b). For precursors **3**–**6**, the products are irregular in appearance (Figure 7c–f). The atomic percentages of the NCs obtained from EDX from all the precursors (**1**–**6**) are shown in Figure S9 and Table S6.

Raman spectroscopy revealed that the MnS synthesized from precursor **1** by solventless thermolysis exhibited a band at 635.18 cm^–1^ which varied insignificantly when using precursors **2**, **3**, **4**, **5**, and **6** (Figure S10 and Table S6), as previously reported.^31^

### MnS Thin Films by Doctor Blading

**Figure 8:**
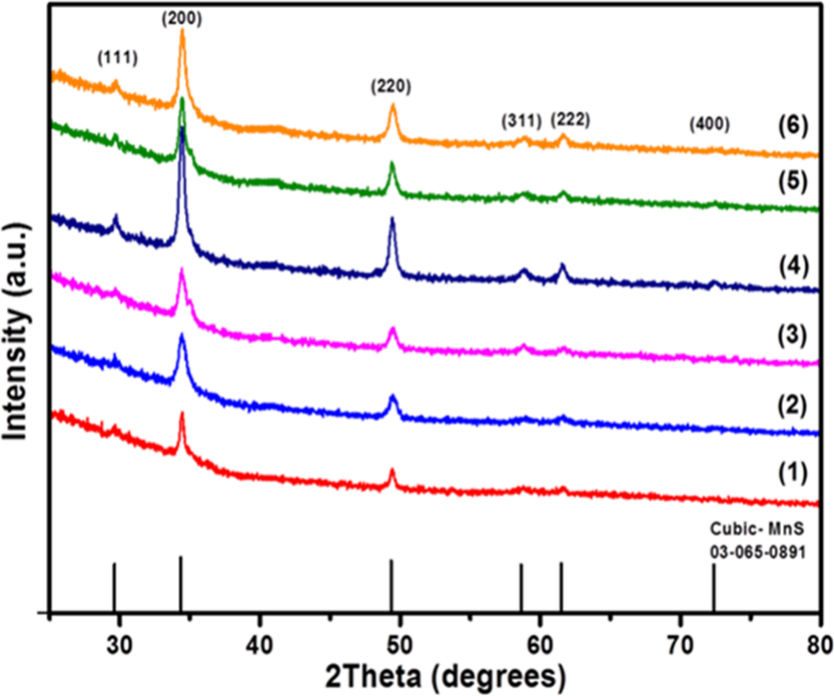
XRD patterns of MnS thin films prepared by the doctor blade method and heated at 350 °C from precursors **1**–**6**. The standard pattern shown is cubic manganese sulfide, MnS (ICDD no. 03-065-0891).

The diffraction peaks of the thin films prepared from complexes **1**–**6** were indexed to cubic manganese sulfide, α-MnS (ICDD # 03-065-0891); see Figure 8. The patterns showed a change in the intensity compared to the bulk pattern that indicated a preferred growth in the (200) and (220) planes at 2θ = 34.4 and 49.4°, respectively, which exists and shows an increase with the precursor chain length. This suggests that the molecular precursor structure influences the nucleation and growth of manganese sulfide thin films under these conditions. Using the Debye–Scherrer equation, the width of the crystallites was found to be 20.8, 14.2, 13.4, 17.6, 16.5, and 16.5 nm for the thin films obtained from the complexes **1**, **2**, **3**, **4**, **5**, and **6**, respectively (Table S7). The sizes of the crystallites are similar to those obtained by using the hot injection method but are larger than those when using the solventless thermolysis. Crystallites that have preferred orientation have been observed in other studies of thin films grown by the chemical bath deposition technique.^32^

**Figure 9:**
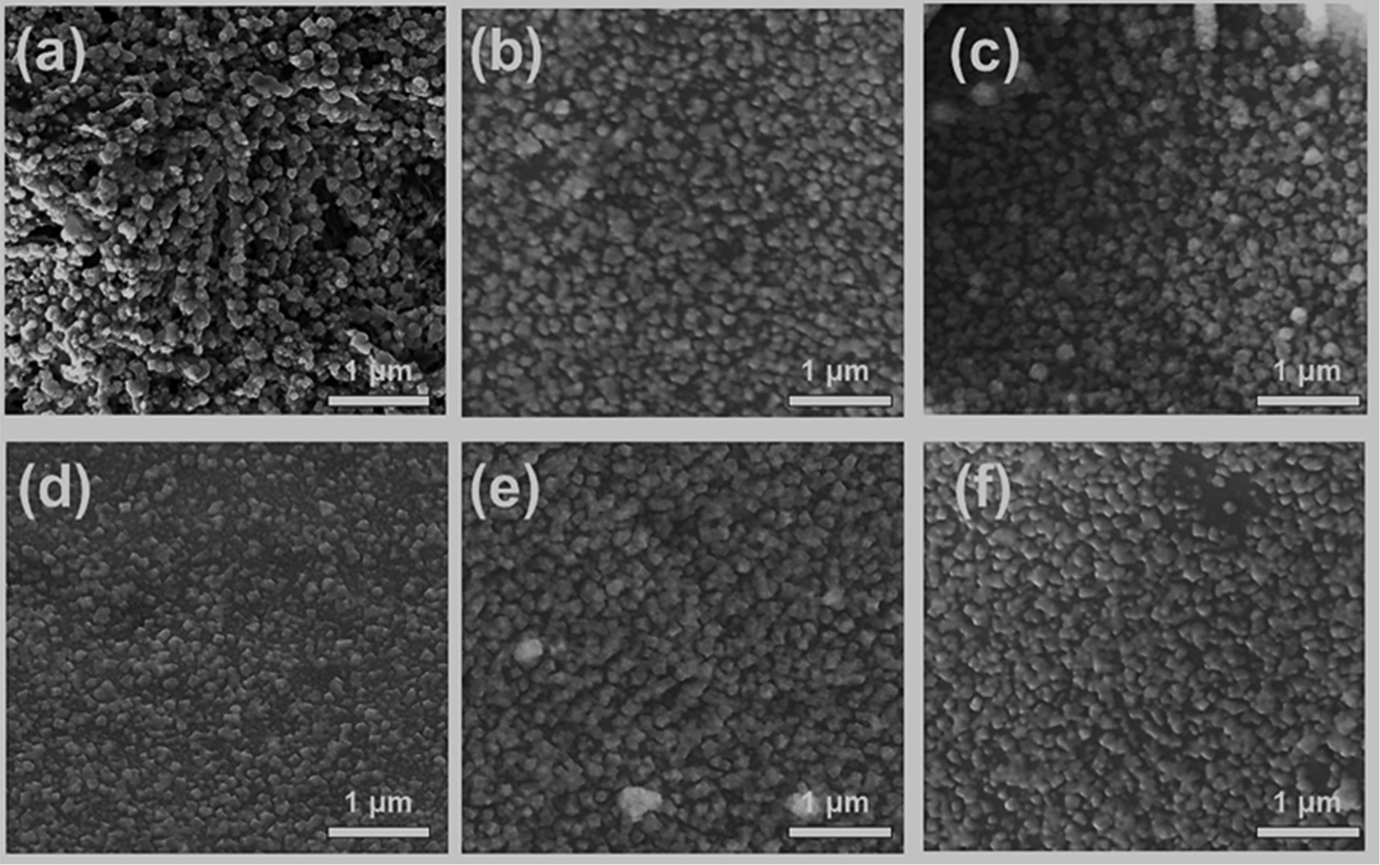
Secondary electron SEM images (10 kV) of MnS thin films using precursors (a–f) **1**–**6** deposited by the doctor blade method at 350 °C.

All precursors resulted in thin films of cubic MnS; see Figure 9a–d. The morphologies of the films from the doctor blade method were significantly different to those from hot injection and solventless thermolysis. EDX spectroscopy revealed the presence of manganese and sulfur in near stoichiometric amounts in all samples (Figures S11 and S7).

Raman spectroscopy revealed that the MnS thin films obtained from the precursors **1**, **2**, **3**, **4**, **5**, and **6** all exhibited bands at approximately 635.89 cm^–1^ (Figure S12 and Table S7) and are in agreement with that of bulk MnS.^31^

### Magnetic Properties of MnS NCs

**Figure 10:**
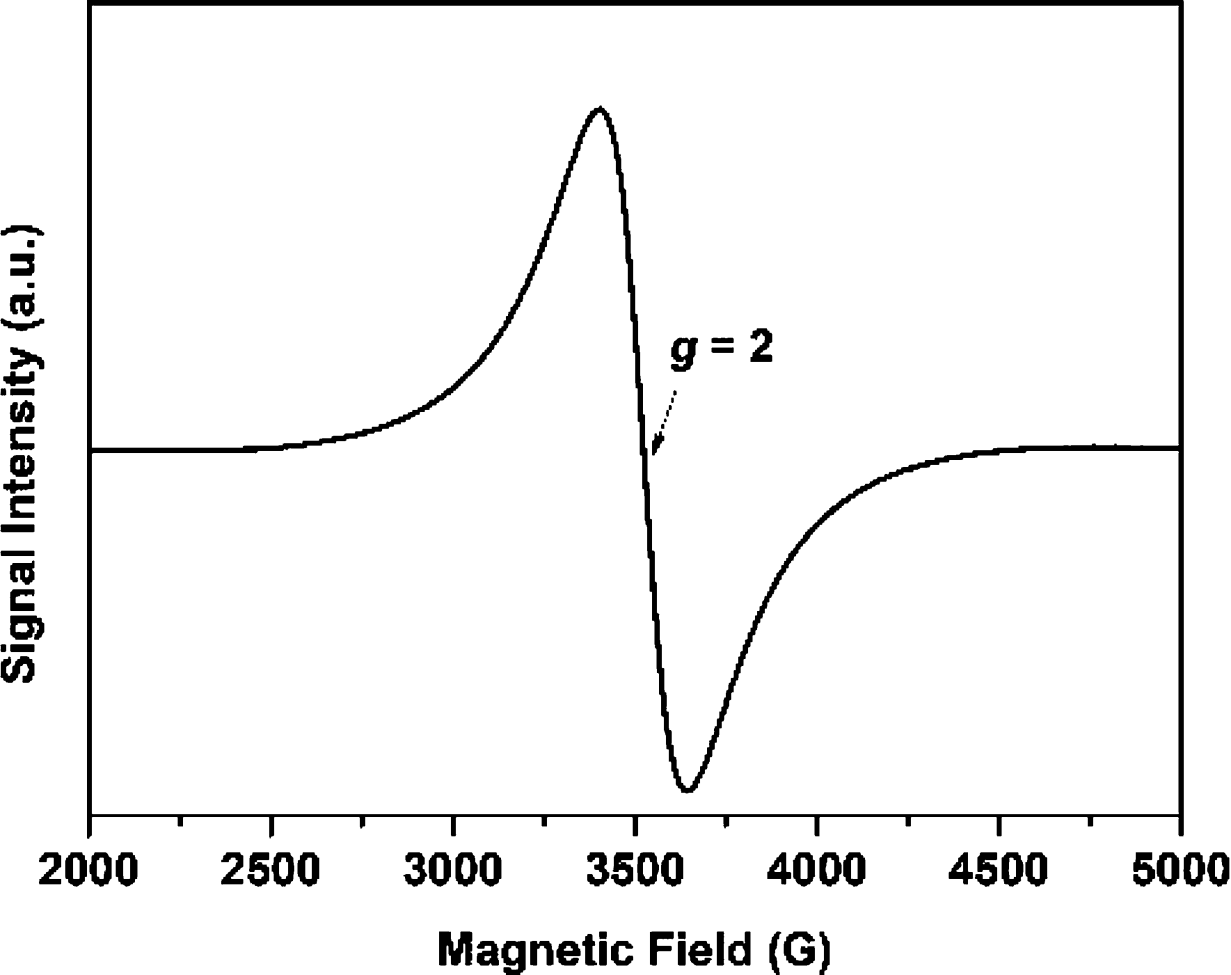
X-band EPR spectrum of α-MnS NCs obtained from complex **2**.

**Figure 11:**
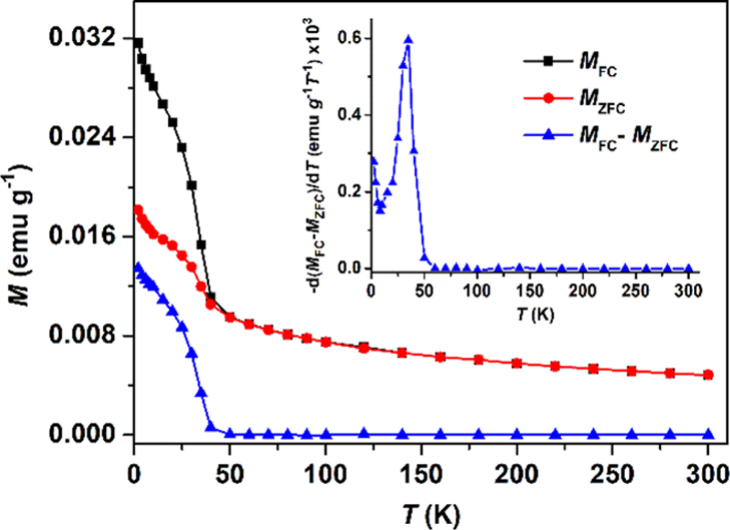
Thermal dependence of magnetization for α-MnS NCs obtained from complex **2**, measured in ZFC (red circles) and FC (black squares) regimes, with the difference *M*_FC_ – *M*_ZFC_ plotted in blue. Inset: plot of −d(*M*_FC_ – *M*_ZFC_)/d*T* for the same NCs.

The magnetic properties of the α-MnS obtained from complex **2** using solventless thermolysis were studied. The room-temperature X-band EPR spectrum of α-MnS NCs obtained from complex **2** displays a strong signal with *g* = 2, characteristic of magnetic NCs (Figure 10). The magnetization of the NCs was measured as a function of temperature, in field-cooled (FC) and zero-field-cooled (ZFC) regimes, under an applied field of 100 Oe (Figure 11). The magnetic properties of α-MnS NCs have been investigated by Kan *et al.*, where at different sizes between 20 and 80 nm, α-MnS NCs were antiferromagnetic (AFM) with reduced interaction strength in smaller NCs. However, these NCs were aggregates with smaller particles, which led to their hysteresis loop being closed.^7^ Puglisi *et al.* reported the magnetic properties of single-crystal octahedral α-MnS NCs of different sizes (14, 20, and 29 nm). Below 50 K, the NCs showed increase in FC magnetization and a maximum ZFC magnetization at 25 K, which both confirmed of a transition between a superparamagnetic (SPM) and ferromagnetic (FM) type.^16^

**Figure 12:**
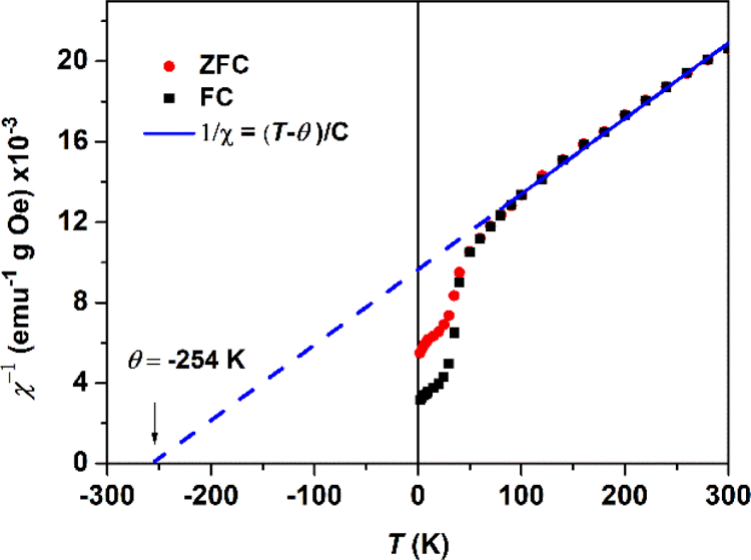
Plot of 1/χ vs temperature for α-MnS NCs obtained from complex **2**, measured in ZFC (red) and FC (black) regimes, with a fit to the Curie law χ = *C*/(*T* – θ) represented in blue (dashed lines).

Irreversible magnetic behavior is observed below 40 K, which marks a transition from the SPM to FM, the latter characterized by blocking of magnetization. The presence of FM-like regions in the material is also evident in the T-dependence of the magnetization difference in Figure 11 (blue triangles). Above 70 K, the ZFC and FC magnetization curves fully superpose and data could be fitted to a Curie–Weiss law, χ = *C*/(*T* – θ), providing a Curie–Weiss constant θ = −254 K (Figure 12).

**Figure 13:**
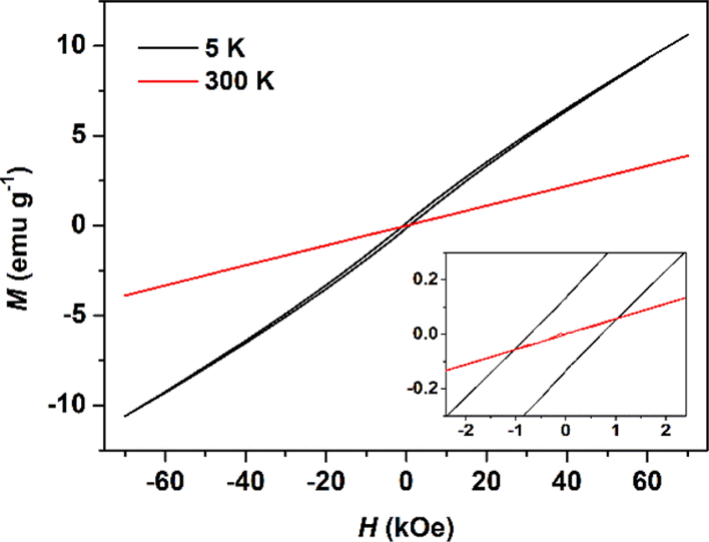
Magnetic hysteresis at 5 and 300 K for α-MnS NCs obtained from **2**. The inset shows the region around zero fields.

The negative sign of θ indicates that the α-MnS NCs obtained from **2** are antiferromagnetic. The θ value is less negative than the bulk value of −465 K^33^ and close to the value that was reported by Puglisi *et al.*, where θ = −272 K for 29 nm.^16^ The AFM interactions become less effective for smaller NCs, approximately in agreement with previous results. The existence of the FM structure at the surface of the α-MnS NCs is additionally supported by the hysteresis measured at 5 and 300 K, as shown in Figure 13. At 300 K, the saturated magnetization was smaller than 5 K, and there were no hysteresis loops. The hysteresis curve recorded at 5 K shows that the magnetization does not saturate up to the magnetic field of 70 kOe, indicative of large anisotropy. Cycling of the magnetization between 70 and −70 kOe reveals a hysteresis loop with a coercive field, *H*_c_, of 0.723 KOe. This field is larger than that observed for similar NCs. Puglisi *et al.* confirmed that α-MnS NC samples obtained by isothermal magnetization at 5 K showed an open loop with size-dependent *H*_c_ of 0.009 kOe (14 nm), 0.081 kOe (20 nm), and 0.180 kOe (29 nm).^16^ Yang *et al.* reported that at low temperature, hysteresis loops were presented in the FM region since they displayed open loops with size-dependent *H*_c_ ranging from 0.01 kOe (14 nm) to 1.265 kOe (40 nm).^3^ This result is, to the best of our knowledge, the first demonstration of a large coercive field (0.723 kOe at 5 K with small size of 6.8 nm) in α-MnS NCs. It is noted that the magnetization of FM materials depends on the size, shape, and structure of these materials.^34^

## Conclusions

The synthesis and the single-crystal X-ray structure of seven novel tetramethylethylenediamine manganese(II) bis(alkylxanthate) complexes in which the alkyl group was methyl (**1**), ethyl (**2**), *n*-propyl (**3**), *n*-butyl (**4**), *n*-pentyl (**5**), *n*-hexyl (**6**), and *n*-octyl (**7**)] were reported. Complexes **2**, **3**, **4**, **6**, and **7** adopted the monoclinic crystal system, while **1** was orthorhombic and **5** was triclinic. All the compounds displayed intermolecular hydrogen bonds through the sulfur atoms of the neighboring molecules (C–H···S), except complex **5**, wherein the (C–H···S) interaction was not observed. The distances of these interactions were slightly shorter than the sum of the contact radii (van der Waals radii). Furthermore, **4** and **5** exhibited intramolecular S–S distances of 3.491 and 3.565 Å, respectively.

Hot injection with OLA lowered the relative decomposition temperatures of the precursors, whereas the solventless thermolysis and the doctor blade technique required a higher decomposition temperature of 350 °C. XRD studies showed that all the precursors broke down cleanly at 250 and 350 °C by all methods to form cubic nanoscale α-MnS with sizes dependent on the breakdown method used. Magnetic measurements revealed that these nanomaterials possess a large coercive field (0.723 kOe for 6.8 nm NCs) in contrast to other studies of nanometric MnS reported in the literature.

## Experimental Section

### Materials and Instrumentation

All chemicals were purchased from Sigma Aldrich or Alfa Aesar and used as received. Melting points were determined with a Stuart melting point apparatus (Cole-Parmer, UK). Infrared spectra (IR) were recorded using a Nicolet iS5 Thermo Scientific ATR instrument in the range 4000–400 cm^–1^ and with a spectral resolution of 4 cm^–1^. Elemental analysis (EA) and TGA were carried out by the Micro-elemental Analysis Service in the Department of Chemistry at the University of Manchester. EA was performed using a Flash 2000 Thermo Scientific elemental analyzer, and TGA data were obtained with a Mettler Toledo TGA/DSC stare system in the range 30–600 °C at a heating rate of 10 °C min^–1^ under nitrogen flow. Powder XRD analyses were carried out using an XPert diffractometer with a Cu Kα1 source (λ = 1.54059 Å), and the samples were scanned between 10 and 80°; the applied voltage and current were 40 kV and 30 mA, respectively. SEM and EDX spectroscopy analysis were carried out using TESCAN MIRA3 FEG-SEM. Raman spectra were measured using a Renishaw 1000 Micro-Raman System equipped with a 514 nm laser. Single-crystal XRD data for all the complexes were obtained using Mo Kα or Cu Kα radiation on a Rigaku FR-X diffractometer. The structures were solved by the SHELXL (Sheldrick, 2015) program.^35^ Non-hydrogen atoms were refined with anisotropic atomic displacement parameters. Hydrogen atoms were placed in calculated positions, assigned isotropic thermal parameters, and allowed to ride on their parent carbon atoms. Crystals suitable for single-crystal XRD were grown using vapor diffusion of hexane into a solution of the requisite metal xanthate in acetone.

### Synthesis of [Mn(S_2_COMe)_2_·(TMEDA)] (**1**)

Potassium methylxanthate was prepared according to the previous publications.^19,25^ In brief, potassium hydroxide (0.76 g, 13.63 mmol) was dissolved in excess methanol and stirred for 2 h at room temperature, followed by cooling to 0 °C. Carbon disulfide (1.04 g, 0.83 mL, 13.63 mmol) was added dropwise to the stirred methanolic solution and stirred for 1 h. An aqueous solution of Mn(CH_3_COO)_2_·4H_2_O (1.6 g, 6.8 mmol) in 50 mL of water was added dropwise to the reaction mixture, which was stirred for 30 min to form a brown/yellow solution. TMEDA (0.79 g, 6.76 mmol) was added to the solution while stirring for 1 h to produce a brown precipitate. The brown precipitate was collected by vacuum filtration and washed with deionized water. The final product was dried *in vacuo* overnight and was subsequently recrystallized from acetone. Yield: 83.5% (3.5 g). mp: 138 °C. Elemental analysis: calc (%): C, 31.17; H, 5.76; S, 33.22; N, 7.27; Mn, 14.27%. Found (%): C, 30.98; H, 5.56; S, 33.22; N, 7.02; Mn, 13.94%. IR (ν_max_/cm^–1^): 2995 (w), 1140–1193 (s), 1037 (s).

### Synthesis of [Mn(S_2_COEt)_2_·(TMEDA)] (**2**)

**2** was prepared as **1** except that excess ethanol was used in place of methanol. Yield: 88.1% (3.7 g). mp: 137 °C. Elemental analysis: calc (%): C, 34.86; H, 6.34; S, 30.96; N, 6.78; Mn, 13.30% (12.2 g). Found (%): C, 34.94; H, 6.28; S, 31.26; N, 6.70; Mn, 13.01%. IR (ν_max_/cm^–1^): 2980 (w), 1142–1185 (s), 1032 (s).

### Synthesis of [Mn(S_2_CO^*n*^Pr)_2_·(TMEDA)] (**3**)

**3** was prepared as **1** except using *n*-propanol in place of methanol. Yield: 77.1% (3.8 g). mp: 134 °C. Elemental analysis: calc (%): C, 38.09; H, 6.86; S, 29.00; N, 6.35; Mn, 12.46%. Found (%): C, 37.88; H, 6.67; S, 29.37; N, 6.12; Mn, 12.18%. IR (ν_max_/cm^–1^): 2968 (w), 1145–1179 (s), 1043 (s).

### Synthesis of [Mn(S_2_CO^*n*^But)_2_·(TMEDA)] (**4**)

**4** was prepared as **1** except using *n*-butanol in place of methanol. Yield: 76.3% (4.1 g). mp: 85 °C. Elemental analysis: calc (%): C, 40.93; H, 7.30; S, 27.26; N, 5.97; Mn, 11.71%. Found (%): C, 40.78; H, 7.15; S, 27.58; N, 5.61; Mn, 11.55%. IR (ν_max_/cm^–1^): 2958 (w), 1043 (s), 1142–1180 (s).

### Synthesis of [Mn(S_2_CO ^*n*^Pent)_2_·(TMEDA)] (**5**)

The complex **5** was prepared as for **1** except using *n*-pentanol in place of methanol. Yield: 78.3% (4.5 g). mp: 65 °C. Elemental analysis: calc (%): C, 43.45; H, 7.70; S, 25.73; N, 5.63; Mn, 11.05%. Found (%): C, 43.41; H, 7.69; S, 25.98; N, 5.42; Mn, 10.86%. IR (ν_max_/cm^–1^): 2952 (w), 1040 (s), 1145–1180 (s).

### Synthesis of [Mn(S_2_CO ^*n*^Hex)_2_·(TMEDA)] (**6**)

**6** was prepared as **1** except using *n*-hexanol in place of methanol. Yield: 82.9% (5.1 g). mp: 63 °C. Elemental analysis: calc (%): C, 45.70; H, 8.06; S, 24.35; N, 5.33; Mn, 10.46%. Found (%): C, 45.30; H, 7.99; S, 24.32; N, 5.01; Mn, 10.20%. IR (ν_max_/cm^–1^): 2952 (w), 1038 (s), 1142–1182 (s).

### Synthesis of [Mn(S_2_CO ^*n*^Oct)_2_·(TMEDA)] (**7**)

**7** was prepared as **1** except using *n*-octanol in place of methanol. Yield: 80.9% (5.5 g). mp: 60 °C. Elemental analysis: calc (%): C, 49.55; H, 8.67; S, 22.00; N, 4.82; Mn, 9.45%. Found (%): C, 49.05; H, 8.42; S, 21.91; N, 4.65; Mn, 9.28%.

### Synthesis of MnS NCs by Hot Injection Thermolysis

The MnS NCs were synthesized by dispersing manganese alkylxanthate (0.2 g) in 2.0 mL of TOP and then injected into 8.0 mL of preheated oleylamine (OLA) at 230 °C with continuous stirring under a nitrogen atmosphere. The temperature was maintained at 230 °C for 30 min, after which the reaction mixture was allowed to cool to room temperature by removal from the heat source. Methanol (12.0 mL) was added to the reaction mixture, and the precipitate was collected by centrifugation.

### Synthesis of MnS NCs by Solventless Thermolysis

A total of 0.4 g of manganese alkylxanthate was placed in a furnace tube under a stream of argon (300 cm^3^ min^–1^). The furnace tube was subsequently heated to 350 °C, held at this temperature for 1 h, and allowed to cool to room temperature.

### Deposition of MnS Films by Doctor Blading

In a typical deposition process, 0.02 g of manganese alkylxanthate was added to 0.2 mL of THF to form a slurry. The as-prepared complex slurry was pasted on a cleaned glass substrate and distributed uniformly on the glass substrates using a doctor blade made up of stainless steel to form wet thin films of MnS. The films were then placed into furnace tube which was then heated to 350 °C for 1 h under a stream of argon (300 cm^3^ min^–1^).

## Supporting Information Available

Crystallographic data for complex **1**(CIF)
Crystallographic data for complex **2** (CIF)
Crystallographic data for complex **3** (CIF)
Crystallographic data for complex **4** (CIF)
Crystallographic data for complex **5** (CIF)
Crystallographic data for complex **6** (CIF)
Crystallographic data for complex **7** (CIF)
Crystallographic data, elemental analysis, infrared spectroscopy, XRD, EDX, Raman, and SEM (PDF)

## Supplementary Material

ao1c02907_si_001.cif

ao1c02907_si_002.cif

ao1c02907_si_003.cif

ao1c02907_si_004.cif

ao1c02907_si_005.cif

ao1c02907_si_006.cif

ao1c02907_si_007.cif
