# Supplementary material for: Structural Investigations of α-MnS Nanocrystals and Thin Films Synthesized from Manganese(II) Xanthates by Hot Injection, Solvent-Less Thermolysis, and Doctor Blade Routes
Source: ACS Omega. 2021 Oct 11;6(42):27716–25. doi: 10.1021/acsomega.1c02907 (PMC8552351; doi:10.1021/acsomega.1c02907)
Supplement: Supplementary file 8 — ao1c02907_si_008.pdf [file ao1c02907_si_008.pdf]

Supporting Information

Structural investigations of α-MnS nanocrystals and thin films synthesised from manganese(II) xanthates by hot injection, solvent-less thermolysis and doctor blade routes.

Abdulaziz M. Alanazi,[†,§] Paul D. McNaughter,[†] Firoz Alam,[†] Inigo J. Vitorica-yrezabal,[†] George F. S. Whitehead,[†] Floriana Tuna,[†] Paul O'Brien[†,‡] David Collison,[†] and David J. Lewis[‡]*

[†] Department of Chemistry, University of Manchester, Oxford Road, Manchester, M13 9PL, UK.

[†] Department of Materials, University of Manchester, Oxford Road, Manchester, M13 9PL, UK

[§] Department of Chemistry, Islamic university, Prince Naif Ibn Abdulaziz Rd, Madinah, 42351, KSA

*Corresponding author: Dr David J. Lewis

E-mail: david.lewis-4@manchester.ac.uk

Table S1: Selected Bond Lengths (Å) and Angles (º) for novel complexes **(1-7)**.

Complexes	*(1)*	*(2)*	*(3)*	*(4)*	*(5)*	*(6)*	*(7)*
				Bond lengths			
Mn-S1	2.5676 (16)	2.5644 (5)	2.6036 (6)	2.5757(6)	2.5853(5)	2.5692 (13)	2.5847 (18)
Mn-S2	2.6543 (15)	2.6750 (4)	2.6188 (5)	2.6179(7)	2.6283(6)	2.6489 (11)	2.6151 (19)
Mn-S3	2.5805 (15)	2.5644 (5)	2.5874 (6)	2.5639(6)	2.5971(5)	2.5692 (13)	2.5847 (18)
Mn-S4	2.5676 (16)	2.6750 (4)	2.6343 (5)	2.6421(7)	2.6094(6)	2.6489 (11)	2.6151 (19)
Mn-N1	2.296 (5)	2.293 (15)	2.3125 (15)	2.316(2)	2.293(2)	2.283 (4)	2.297 (5)
Mn-N2	2.295 (4)	2.293 (15)	2.3050 (16)	2.304(2)	2.341(2)	2.283 (4)	2.297 (5)
C-O	1.331 (6)	1.333 (2)	1.334 (4)	1.334 (6)	1.334 (4)	1.361 (5)	1.341 (9)
	1.325 (4)		1.335 (3)	1.332 (4)	1.327 (6)		
				Bond Angles			
S1-Mn-S2	69.41 (4)	69.10 (15)	69.52 (16)	69.46(2)	69.59(2)	69.21 (4)	69.35 (8)
S3-Mn-S4	69.12 (4)	69.10 (15)	69.24 (16)	69.56(2)	69.42(2)	69.21 (4)	69.35 (8)
N1-Mn-N2	78.58 (18)	79.22 (8)	78.90 (6)	79.16(8)	79.31(5)	78.3 (3)	78.49 (3)
S1-Mn-N1	93.34(1)	92.56 (4)	94.10(4)	96.15(6)	92.33(4)	92.95 (14)	96.77 (15)
S2-Mn-N1	102.24 (13)	104.16 (4)	102.44 (4)	100.29(6)	99.59(4)	103.23 (11)	101.91 (16)
S3-Mn-N2	93.54 (13)	92.56 (4)	94.79(4)	95.32(6)	92.44(4)	92.95 (14)	96.77 (15)
S4-Mn-N2	103.30(1)	104.16 (4)	98.85 (4)	102.00 (6)	98.56(4)	103.23 (11)	101.91 (16)

Table S2: X-ray crystallographic data and refinement details for (**1**-**7**) using Cu Kα radiation and with H-atom parameters constrained.

Complex	***(1)***	***(2)***	***(3)***	***(4)***	***(5)***	***(6)***	***(7)***
Chemical formula	$C_{10}H_{22}MnN_2O_2S_4$	$C_{12}H_{26}MnN_2O_2S_4$	$C_{14}H_{30}MnN_2O_2S_4$	$C_{16}H_{34}MnN_2O_2S_4$	$C_{18}H_{38}MnN_2O_2S_4$	$C_{20}H_{42}MnN_2O_2S_4$	$C_{24}H_{50}MnN_2O_2S_4$
M_r	385.47	413.53	441.58	469.63	497.68	525.73	581.84
Crystal system, space group	Orthorhombic, *Pbca*	Monoclinic, *C*2/*c*	Monoclinic, $P2_1/c$	Monoclinic, $P2_1/c$	Triclinic, $P\bar{1}$	Monoclinic, *I*2/*a*	Monoclinic, *I*2/*a*
Temperature (K)	293	100	100	150	100	240	150
a, (Å)<br>***b, (Å)***<br>***c, (Å)***	15.2336 (8)<br>16.3399 (7)<br>13.8528 (7)	20.8959 (13)<br>8.0893 (4)<br>15.3732 (9)	11.8338 (5)<br>11.9042 (4)<br>15.5003 (6)	12.5433 (3)<br>21.2751 (5)<br>9.3308 (2)	7.5898 (4)<br>11.8599 (5)<br>16.0804 (7)	15.6601 (3)<br>8.15454 (13)<br>23.5223 (5)	13.8999 (11)<br>8.5486 (6)<br>27.052 (3)
α (°)<br>***β (°)***<br>***γ (°)***	90<br>90<br>90	90<br>132.491 (7)<br>90	90<br>106.751 (2)<br>90	90<br>102.885 (2)<br>90	70.704 (4),<br>78.932 (4),<br>72.591 (4)	90<br>108.507 (2)<br>90	90<br>97.371 (10)<br>90
V (Å³)	3448.2 (3)	1916.1 (2)	2090.90 (14)	2427.32 (10)	1296.60 (11)	2848.48 (10)	3187.9 (5)
Z	8	4	4	4	2	4	4
μ (mm⁻¹)	10.75	9.71	8.94	7.73	7.26	6.64	5.98
Crystal size (mm)	0.3 × 0.1 × 0.04	0.22 × 0.14 × 0.06	0.24 × 0.13 × 0.06	0.16 × 0.11 × 0.01	0.20 × 0.07 × 0.01	0.46 × 0.31 × 0.02	0.4 × 0.35 × 0.1
T_{min}, T_{max}	0.282, 1.000	0.504, 0.593	0.469, 0.616	0.353, 1.000	0.743, 1.000	0.445, 1.000	0.664, 1.000
No. of measured, independent and observed [$I > 2\sigma(I)$] reflections	15125, 3288, 2271	6616, 1868, 1670	16722, 4099, 3538	11697, 4724, 3860	14258, 4999, 4661	29808, 2598, 2422	10324, 3236, 2083
R_{int}	0.096	0.045	0.053	0.039	0.034	0.042	0.065
$(\sin \theta/\lambda)_{max}$ (Å⁻¹)	0.623	0.617	0.617	0.617	0.617	0.602	0.628
$R[F^2 > 2\sigma(F^2)]$, $wR(F^2)$, S	0.070, 0.223, 1.05	0.026, 0.063, 1.05	0.028, 0.066, 1.02	0.037, 0.101, 1.06	0.031, 0.079, 1.06	0.068, 0.183, 1.10	0.099, 0.334, 1.13
No. of reflections	3288	1868	4099	4724	4999	2598	3236
No. of parameters	178	99	214	291	250	191	180
$\Delta\rangle_{max}$, $\Delta\rangle_{min}$ (e Å⁻³)	1.78, -0.73	0.34, -0.22	0.34, -0.24	0.54, -0.45	0.30, -0.47	1.34, -0.45	0.66, -0.80

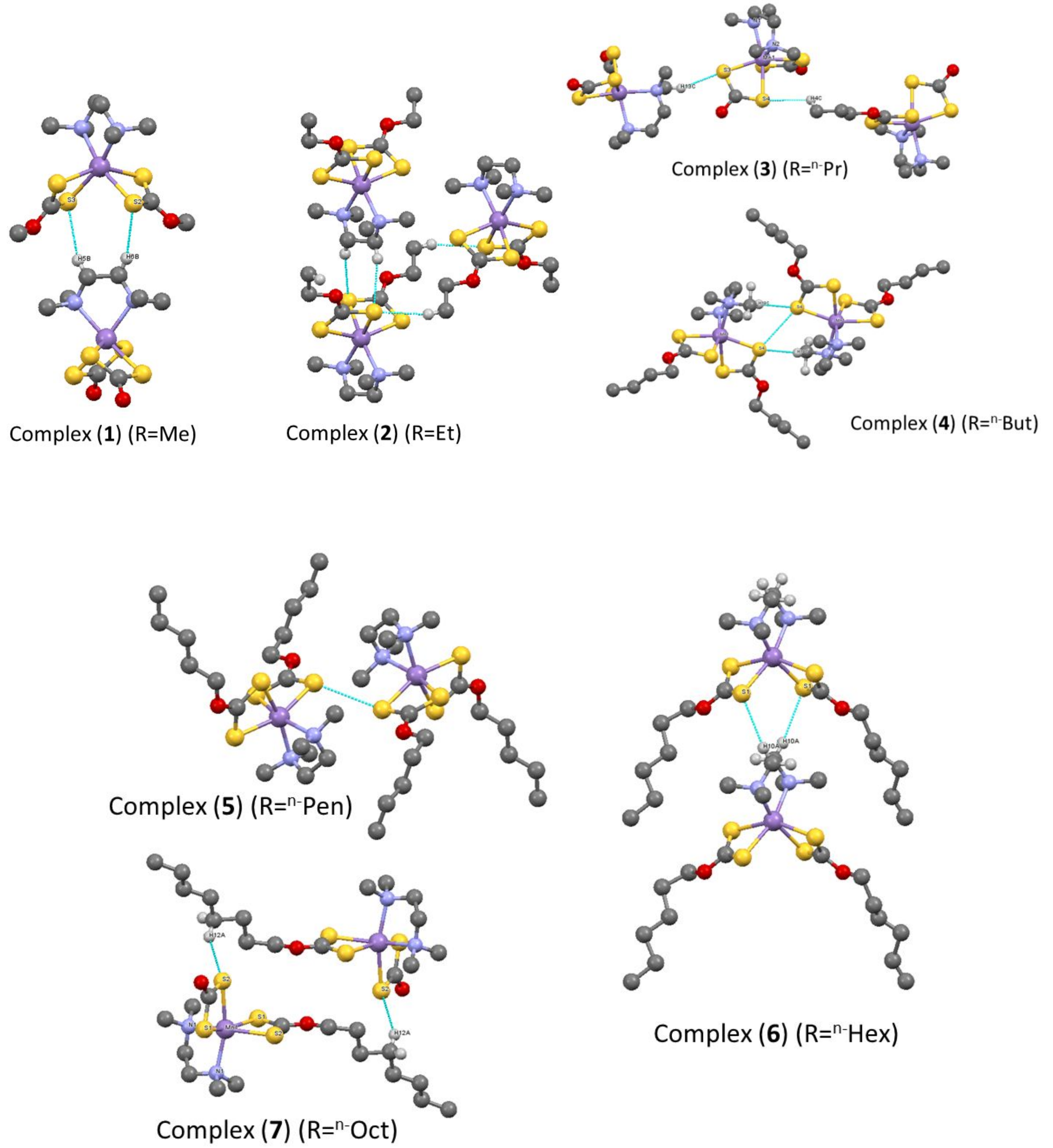

Figure S1. Crystal structures of **1**, **2**, **3**, **4**, **5**, **6** and **7** showing intermolecular C–H···S non-covalent contacts and S···S interactions.

Table S3: Details of selected intermolecular non-covalent contacts (Å) in the prepared compounds **(1-7)**.

Complexes	***N–C–H···S interactions distance***	***C–C–H···S interactions distance***
1	**2.886** [a)]	**-**
2	**2.840** [a)]	**2.842** [a)]
3	**2.894** [a)]	**2.970** [a)]
4	**2.997** [a)]	**-**
5	**-**	**-**
6	**2.927** [a)]	**2.885** [a)]
7	**-**	**2.756** [a)]

[a)] Sum of the contact radii = 3.00 [53]

(53) Bondi, A. "Van Der Waals Volumes and Radii." *J. Phys. Chem.* 1964, 68 (3), 441–451. https://doi.org/10.1021/j100785a001.

Table S4: Elemental and thermal analyses of complexes **1 - 7**.

Complexes	*Elements analysis : Calc (found) %*					*M. Pt (^{o}C)*	*Temperature of TGA (^{o}C)*	*Mass loss (%)*
	**C**	**H**	**S**	**N**	**Mn**			**(Calc.) Found**
(1)	31.17 (30.98)	5.76 (5.56)	33.22 (33.22)	7.27 (7.02)	14.27 (13.94)	138	200 - 350	(22.6) 24.9
(2)	34.86 (34.94)	6.34 (6.28)	30.96 (31.26)	6.78 (6.70)	13.30 (13.01)	137	200 - 350	(21.1) 24.4
(3)	38.09 (37.88)	6.86 (6.67)	29.00 (29.37)	6.35 (6.12)	12.46 (12.18)	134	200 - 350	(19.7) 20.5
(4)	40.93 (40.78)	7.30 (7.15)	27.26 (27.58)	5.97 (5.61)	11.71 (11.55)	85	200 - 350	(18.5) 19.9
(5)	43.45 (43.41)	7.70 (7.69)	25.73 (25.98)	5.63 (5.42)	11.05 (10.86)	65	200 - 350	(17.5) 17.77
(6)	45.70 (45.30)	8.06 (7.99)	24.35 (24.32)	5.33 (5.01)	10.46 (10.20)	63	200 - 350	(16.5) 17.36
(7)	49.55 (49.05)	8.67 (8.42)	22.00 (21.91)	4.82 (4.65)	9.45 (9.28)	60	-	-

Infra-red spectroscopy

The Figure 3 shows the FTIR spectra of all the complexes. The *v*(C=S) and *v*(C–O–C) are the two important bands of the xanthate moiety because the additional π–electron flows from the oxygen atom to the sulphur atoms *via* a planar delocalized π–orbital system. The IR spectra of the as-synthesized complexes $[Mn(S_2COMe)_2.TMEDA]$ **(1)**, $[Mn(S_2COEt)_2.TMEDA]$ **(2)**, $[Mn(S_2CO^nPr)_2.TMEDA]$ **(3)**, $[Mn(S_2CO^nBut)_2.TMEDA]$ **(4)**, $[Mn(S_2CO^nPen)_2.TMEDA]$ **(5)** and $[Mn(S_2CO^nHex)_2.TMEDA]$ **(6)** revealed that the *v*(C=S) vibration was at approximately 1034–1046 cm^{-1}, while the band around 1140–1190 cm^{-1} was attributable to the stretching vibrations of the *v*(C–O–C) asymmetric group, as shown in Figure 3. Moreover, as reported by

Bonati and Ugo *et al.* for analogous dithiocarbamate complexes, the *v*(C–S) stretching frequencies may be used to distinguish between the monodentate and the bidentate behaviours of the 1,1-dithiolate ligands. In the case of monodentate dithiolate ligands, a doublet peak appeared around 1000 cm^{-1} separated by ≥20 cm^{-1}, which could be attributed to the non-equivalence of two C=S stretching vibrations.[54] In contrast, in the case of bidentate dithiolate ligands, a strong singlet was observed in the ∼1000 cm^{-1} region, which was indicative of a symmetrically bound dithiolate moiety. In the present series of manganese complexes, we observed only one strong band at approximately 1030 cm^{-1}, which indicated that all the xanthate ligands were bidentate and symmetrically bonded.

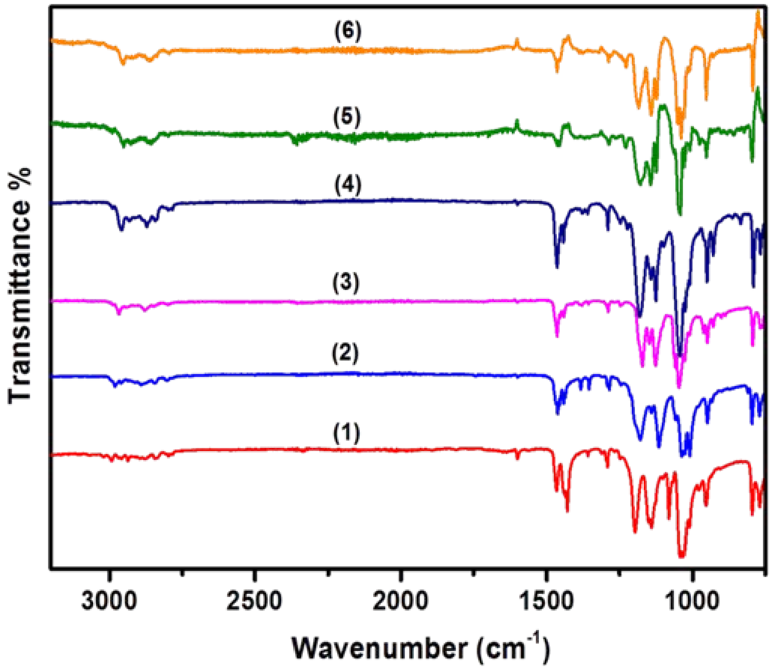

Figure S2: IR spectra of manganese alkyl xanthate precursors **(1-6)**.

Manganese sulphide nanoparticles by the hot injection method:

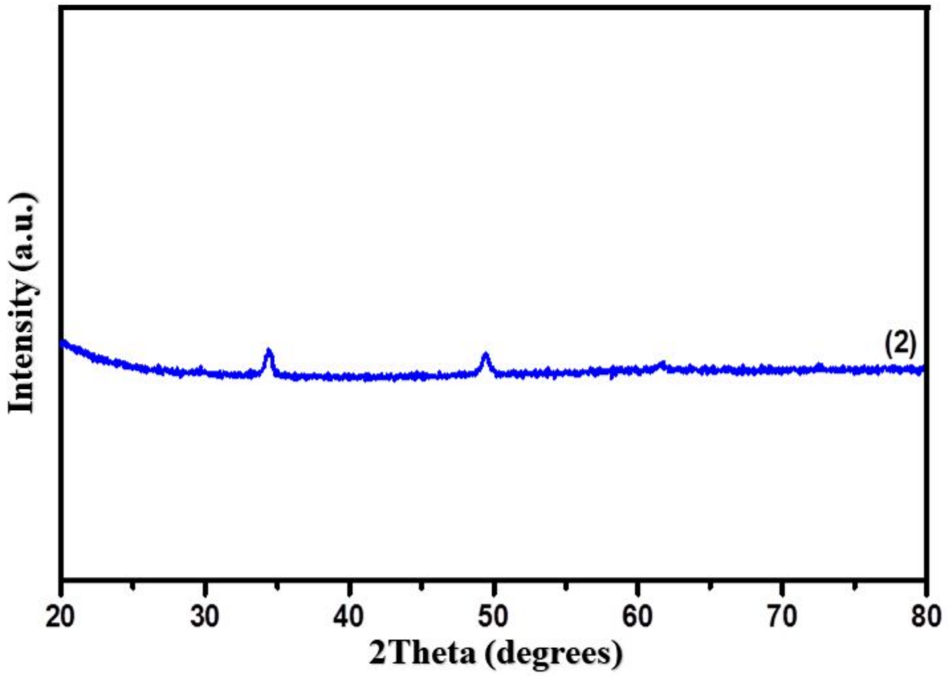

Figure S3: The XRD patterns of manganese sulphide nanoparticles prepared by hot-injection from [Mn(S_2COEt)$_2$(TMEDA)] **(2)** complex heated at different temperature 200 °C for 30 min to determine the optimum temperature for thermal decomposition.

Table S5: The unit cell parameters for the MnS synthesised by hot injection method from precursors **(1-6)**, with (ICDD No. 03-065-0891) as the MnS reference pattern, volume, crystallite size, EDX measurements and Raman data from these samples.

MnS from Complexes	Lattice constant *a* (Å)	Volume ($Å^3$)	Crystallite size (nm)	EDX measurements		Raman shift (cm^{-1})
				Mn (at%)	S (at%)	
(1)	5.214	141.75	19.5 ± 1.45	48.82	51.18	635.89
(2)	5.216	141.91	17.8 ± 1.14	48.65	51.35	636.21
(3)	5.227	141.99	17.0 ± 1.02	48.73	51.27	635.89
(4)	5.220	142.24	14.9 ± 0.98	49.01	50.99	635.90
(5)	5.221	142.32	10.0 ± 0.87	48.93	51.07	635.87
(6)	5.224	142.56	9.18 ± 0.80	48.32	51.68	634.88

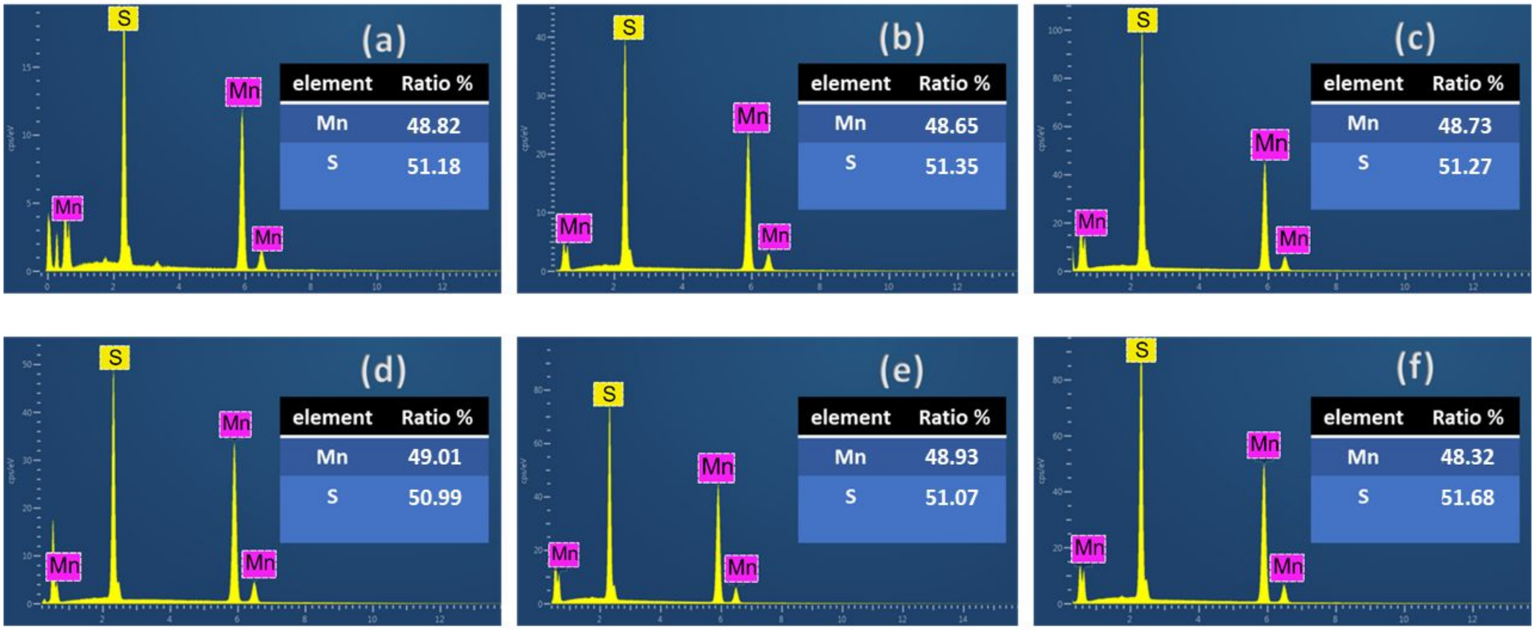

Figure S4: EDX spectra from MnS from precursors (**a-f**) (**1-6**) prepared by hot injection method.

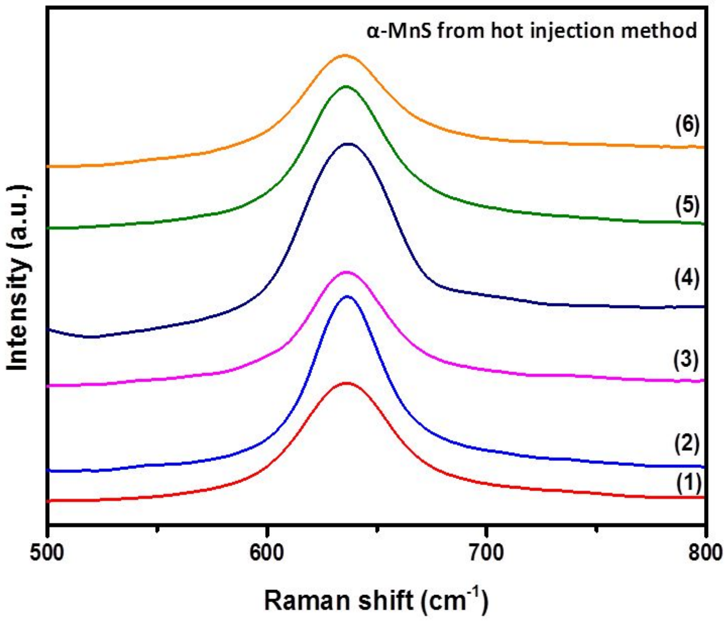

Figure S5: Raman spectra of cubic rock-salt α-MnS from complexes (**1-6**) synthesised by hot injection method.

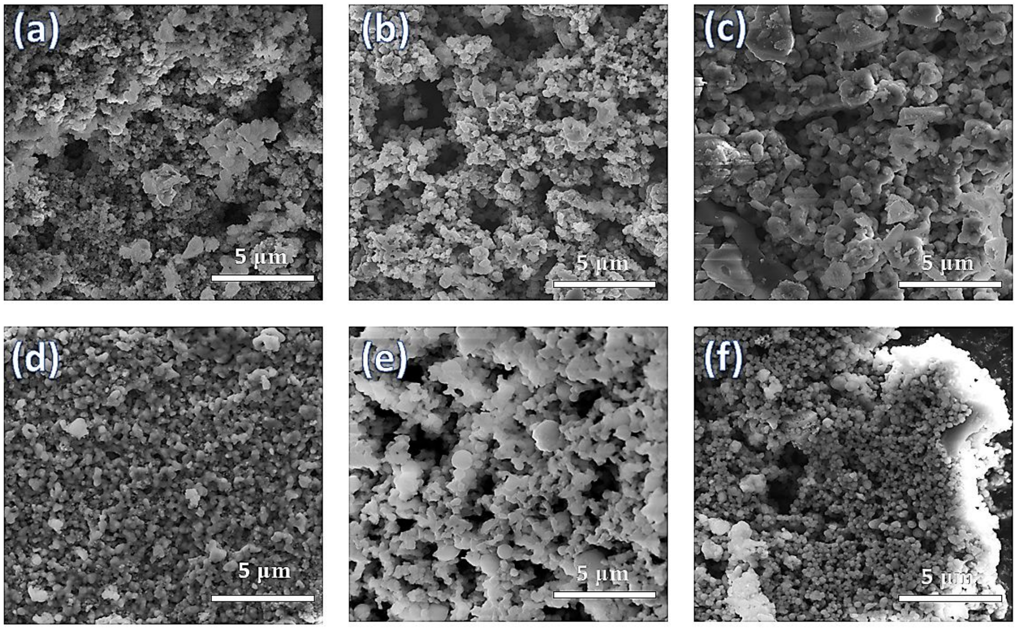

Figure S6: SEM images of MnS nanoparticles from complex (**a-f**) (**1-6**) prepared by hot injection method at 250 °C.

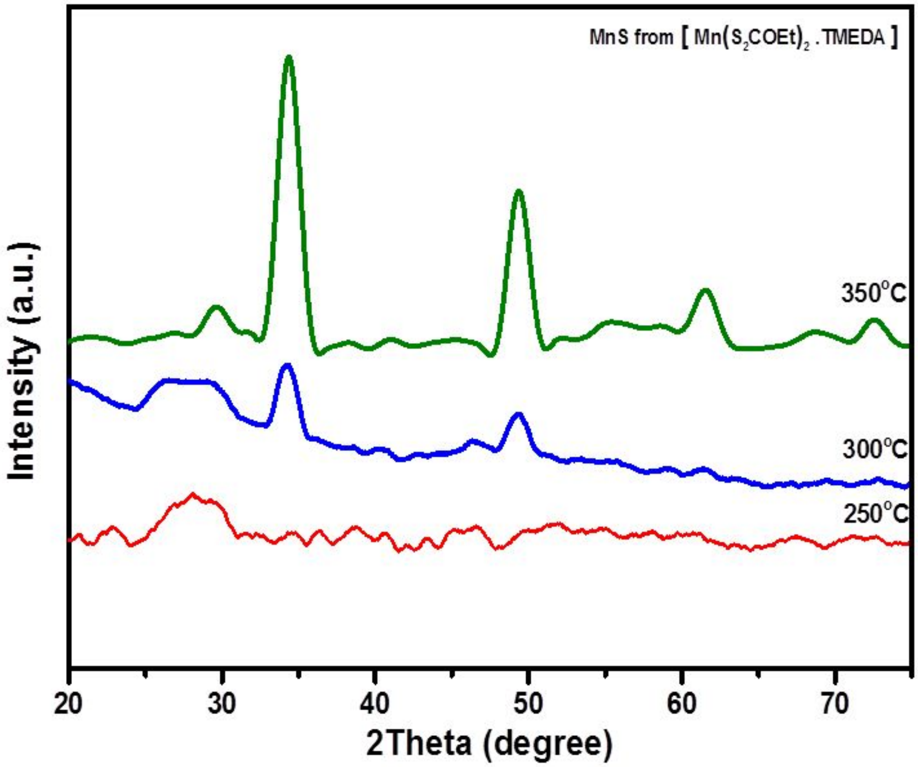

Figure S7: The XRD patterns of manganese sulphide nanoparticles prepared by melt method from $[Mn(S_2COEt)_2(TMEDA)]$ (**2**) complex heated at different temperature 250, 300 and 350 °C for 60 min to determine the optimum temperature for thermal decomposition.

Table S6: The unit cell parameters for the MnS synthesised by melt method from precursors **(1 – 6)**, with (ICDD No. 03-065-0891) as the MnS reference pattern, volume, crystallite size and EDX measurements from these samples.

MnS from Complexes	Lattice constant *a* (Å)	Volume (Å^3)	Crystallite size (nm)	EDX measurements		Raman shift (cm^{-1})
				Mn (at%)	S (at%)	
(1)	5.225	142.65	8.2 ± 0.87	48.44	51.56	635.18
(2)	5.219	142.15	6.8 ± 0.64	48.72	51.28	635.89
(3)	5.223	142.48	6.3 ± 0.98	48.59	51.41	637.02
(4)	5.211	142.50	8.9 ± 1.22	49.27	50.73	636.21
(5)	5.223	142.48	7.6 ± 1.03	50.08	49.92	633.98
(6)	5.210	142.42	8.7 ± 1.15	48.68	51.32	634.52

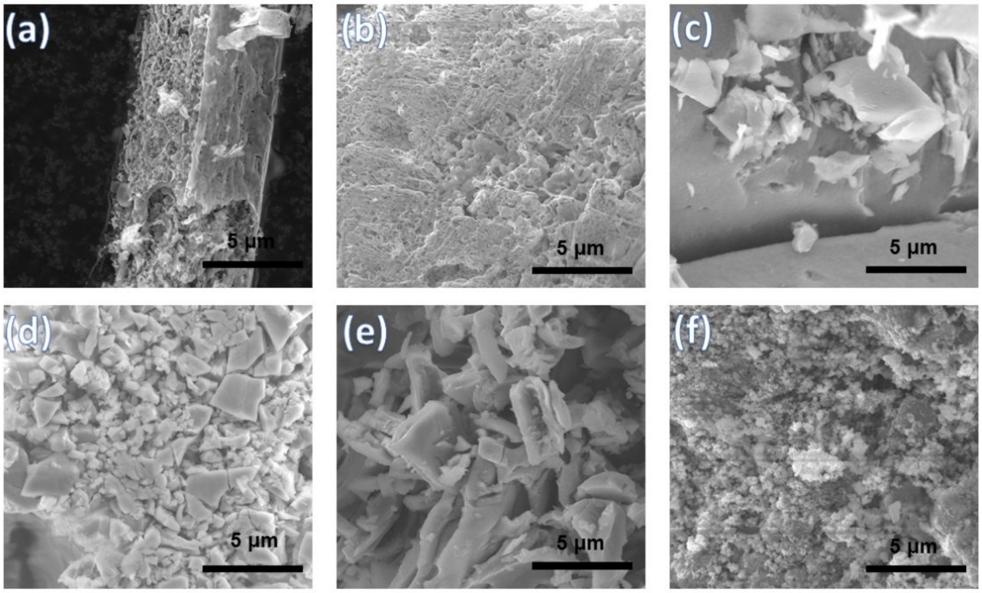

Figure S8: SEM images of MnS nanoparticles from complex (**a-f**) (**1-6**) prepared by melt method at 350 °C.

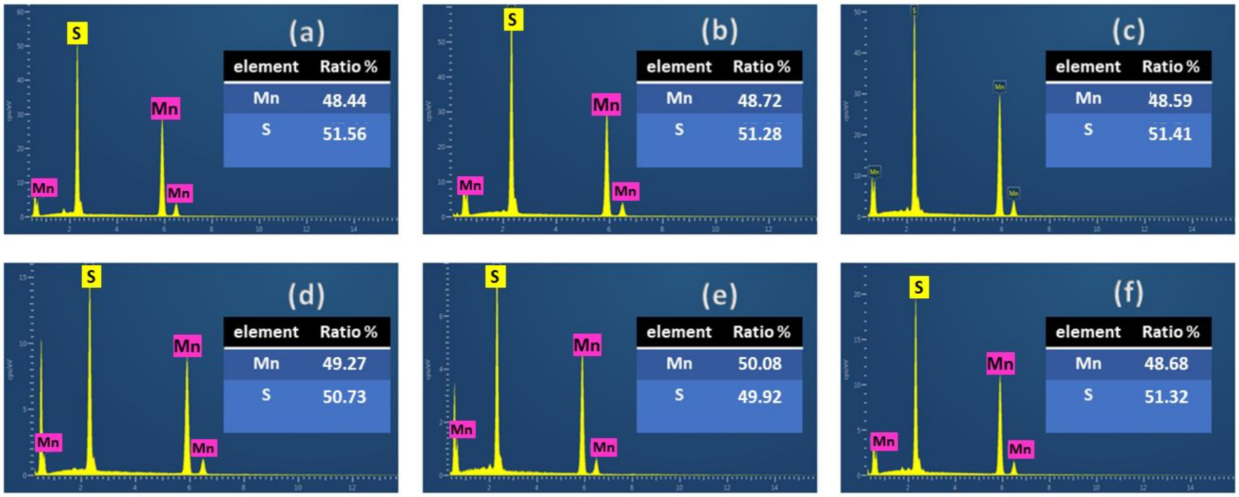

Figure S9: EDX spectra from MnS from precursors (**a-f**) (**1** – **6**) prepared by melt method.

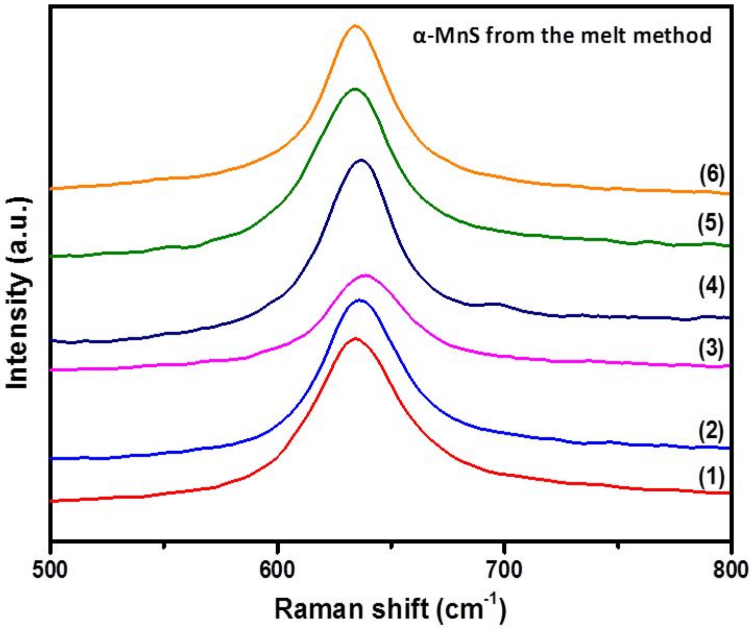

Figure S10: Raman spectra of cubic rock-salt α-MnS from complexes (**1-6**) synthesised by melt method.

Table S7: The unit cell parameters for the MnS synthesised by doctor blade method from precursors (**1-6**), with (ICDD No. 03-065-0891) as the MnS reference pattern, volume, crystallite size and EDX measurements from these samples.

**MnS from Complexes**	**Lattice constant *a* (Å)**	**Volume (Å^3)**	**Crystallite size (nm)**	**EDX measurements**		**Raman shift (cm^{-1})**
				**Mn (at%)**	**S (at%)**	
(1)	5.20	140.61	20.8 ± 1.20	51.89	48.11	635.89
(2)	5.22	142.24	14.2 ± 0.87	51.38	48.62	636.60
(3)	5.20	140.61	13.4 ± 1.12	50.23	49.77	636.21
(4)	5.22	142.24	17.6 ± 1.41	50.36	49.64	635.18
(5)	5.21	142.42	16.5 ± 1.18	50.60	49.40	635.89
(6)	5.22	142.24	16.5 ± 1.17	50.34	49.66	636.92

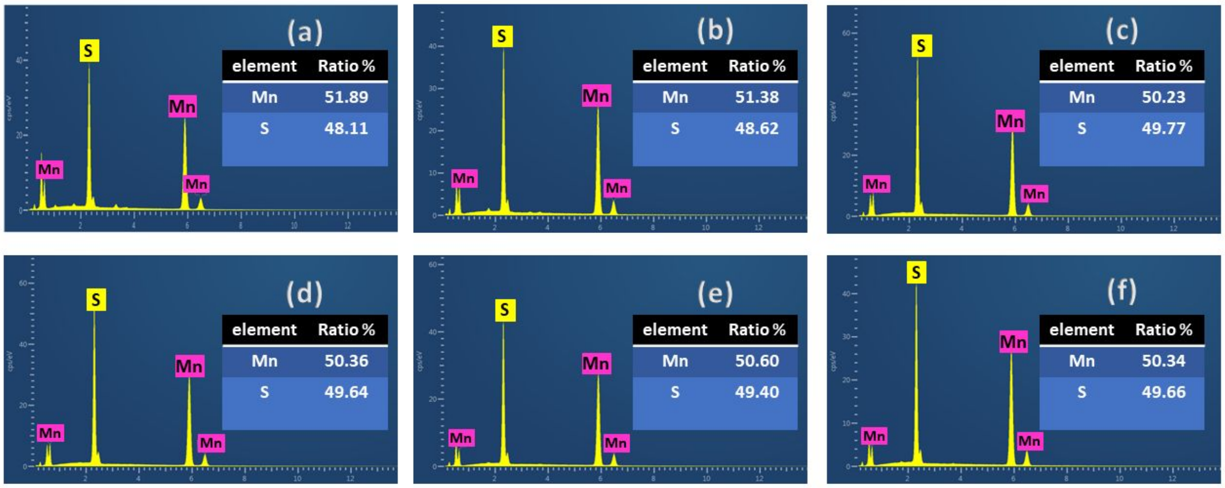

Figure S11: EDX spectra from MnS thin films from precursors (**a-f**) (**1-6**) prepared by doctor blade method.

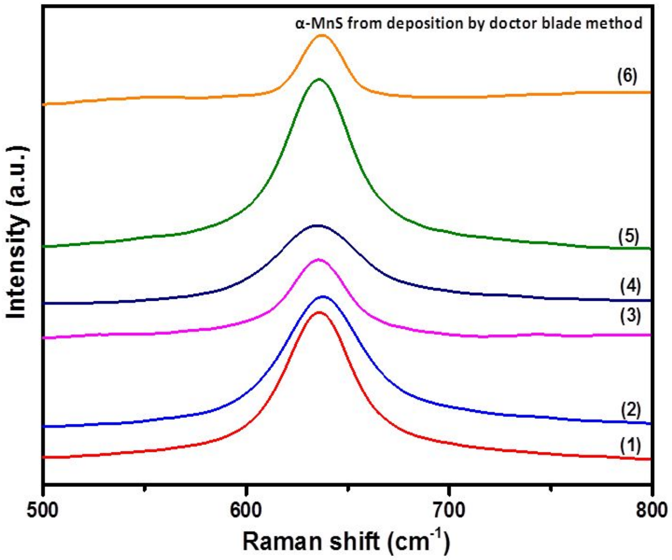

Figure S12: Raman spectra of cubic rock-salt (RS) α-MnS from complexes (**1-6**) Deposition by the doctor blade method.
